# Effect of Concentrated Growth Factor (CGF) on the Promotion of Osteogenesis in Bone Marrow Stromal Cells (BMSC) *in vivo*

**DOI:** 10.1038/s41598-018-24364-5

**Published:** 2018-04-12

**Authors:** Xia Chen, Jian Wang, Li Yu, Jia Zhou, Danning Zheng, Bo Zhang

**Affiliations:** Department of Plastic and Reconstructive Surgery, Shanghai Ninth People’s Hospital, Shanghai Jiao Tong University School of Medicine, 639 Zhizaoju Road, Shanghai, 200011 China

## Abstract

The therapeutic method traditionally used in bone defect reconstruction is autologous bone grafting. The most common problems affecting this type of repair approach are bone absorption and donor trauma. The approach taken in this study overcomes these problems. Bone marrow stromal cells (BMSCs) provided the crucial seed cells. Fibrin biological scaffolds were formed by combining the BMSCs with concentrated growth factor (CGF). BMSCs were isolated from Wistar rat femurs; CGF was prepared from rat heart blood. Five repair groups were created for comparative purposes: (A) CGF + BMSCs; (B) CGF; (C) collagen + BMSCs; (D) collagen; (E) blank. After three months, the rats were sacrificed, and histopathology and three-dimensional CT images produced. Bone regeneration was significantly higher in the (A) CGF + BMSC group; osteogenesis was lower in the (B) CGF and (C) collagen + BMSC groups, at very similar levels; the (D) collagen and (E) blank groups scored the lowest results. Our research suggests that combining CGF with BMSCs leads to the formation of fibrin scaffolds that have a powerful effect on osteogenesis as well as a subsidiary angiogenic effect. SEM images of the CGF scaffolds cultured with BMSCs confirmed good CGF biocompatibility. The superior osteoinductive activity of the CGF + BMSC combination makes it an excellent biomaterial for bone regeneration.

## Introduction

A great deal of bone defect repair research and clinical study has focused on therapeutic approaches to improve the survival rate of bone marrow grafting^1^; less attention has been paid to the study of bone regeneration. However, bone grafts have limitations. Bone absorption and apoptosis is inevitable. One year after operation, Tai^2^ and Van der Meij^3^ respectively reported absorption rates of 43.1% and 31.0%. In addition, the donor sites suffer an extra trauma which sometimes gives rise to complications^4^.

To counter these disadvantages, researchers have tried several strategies. Many researchers have studied the effectiveness of stem cells in bone regeneration^5–7^. Kuznetsov^8^ found that bone marrow derived stromal cells played a critical role in long-term mandibular bone augmentation. Some studies showed platelet-rich plasma (PRP) promoted osteogenesis because it was rich in growth factors^9–11^. More recent studies claimed that better bone regeneration effects could be obtained by combining stem cells with PRP^12,13^. The disadvantage of PRP is its complex preparation process: several procedures are needed to isolate it and it has to be activated by thrombin and calcium chloride.

In research carried out in 2013, Honda *et al*. carried out bone tissue regeneration experiments on rat calvaria defects using a CGF + BMSC combination and signaled excellent healing of a critical-size bone defect *in vivo*. Our results reinforce and extend these findings. Since our primary concerns were biocompatibility and possible BMSC osteogenesis interference, we opted to use a different culture method, a wider range of experimental groups and made scanning electron microscopy (SEM) images of the CGF scaffolds cultured with BMSCs. These images confirmed very positive CGF biocompatibility properties.

CGF (concentrated growth factor), the third-generation platelet concentrate introduced by Sacco in 2006^14^, contains more growth factors and has a harder fibrin structure than first-generation PRP and second-generation PRF (platelet-rich fibrin)^15^. Our team combined CGF with BMSCs for *in vivo* bone defect repair.

## Materials and Methods

### Isolation and culture of Bone Marrow Stromal Cells

All animal operations were performed strictly in accordance with NIH guidelines and ethical principles for the care and use of laboratory animals approved by the Animal Research Committee of Shanghai Ninth People’s Hospital affiliated to Shanghai Jiao Tong University, School of Medicine. All experimental animals were managed humanely. The animals were housed in 12-hour light/dark cycles, in an air-conditioned environment (22 ± 2 °C) and given free access to food pellets and water. Eight-week-old Wistar rats were sacrificed and bilateral femurs were harvested under aseptic conditions. Both ends of the femurs were cut off and the bone marrow was flushed out using a 25-gauge needle with DMEM (Hyclone, Logan City, UT) supplemented with 10% fetal bovine serum (FBS, Gibco, USA), 100 U/ml penicillin and 100 μg/ml streptomycin. The primary BMSCs were cultured in a humidified 37 °C and 5% CO_2_ incubator and the medium was renewed every three days. The cells were passed with 0.25% trypsin (Hyclone) till they reached 90% confluence. BMSCs cultured to the third passages were used in this study.

### Flow cytometry identification of BMSCs

BMSCs were identified by flow cytometry analysis of cell surface markers. BMSCs were digested using 0.25% trypsin to a concentration of 1 × 10^6^ cells/ml. Cell suspensions to which antibodies had been added served as experimental groups: CD9 (BD), CD29 (BD), CD31 (BD), CD34 (BD), CD45 (Biolegend) and CD90 (Biolegend); those without antibodies were controls. After being incubated in a dark place at 4 °C for one hour with phycoerythrin (PE) or fluorescein isothiocyanate (FITC) and then washed with PBS, the labeled BMSCs were resuspended for analysis using a flow cytometer (Epics Altra, USA).

### Fabrication and characterization of concentrated growth factor extracts and fibrin scaffolds

**Figure 1:**
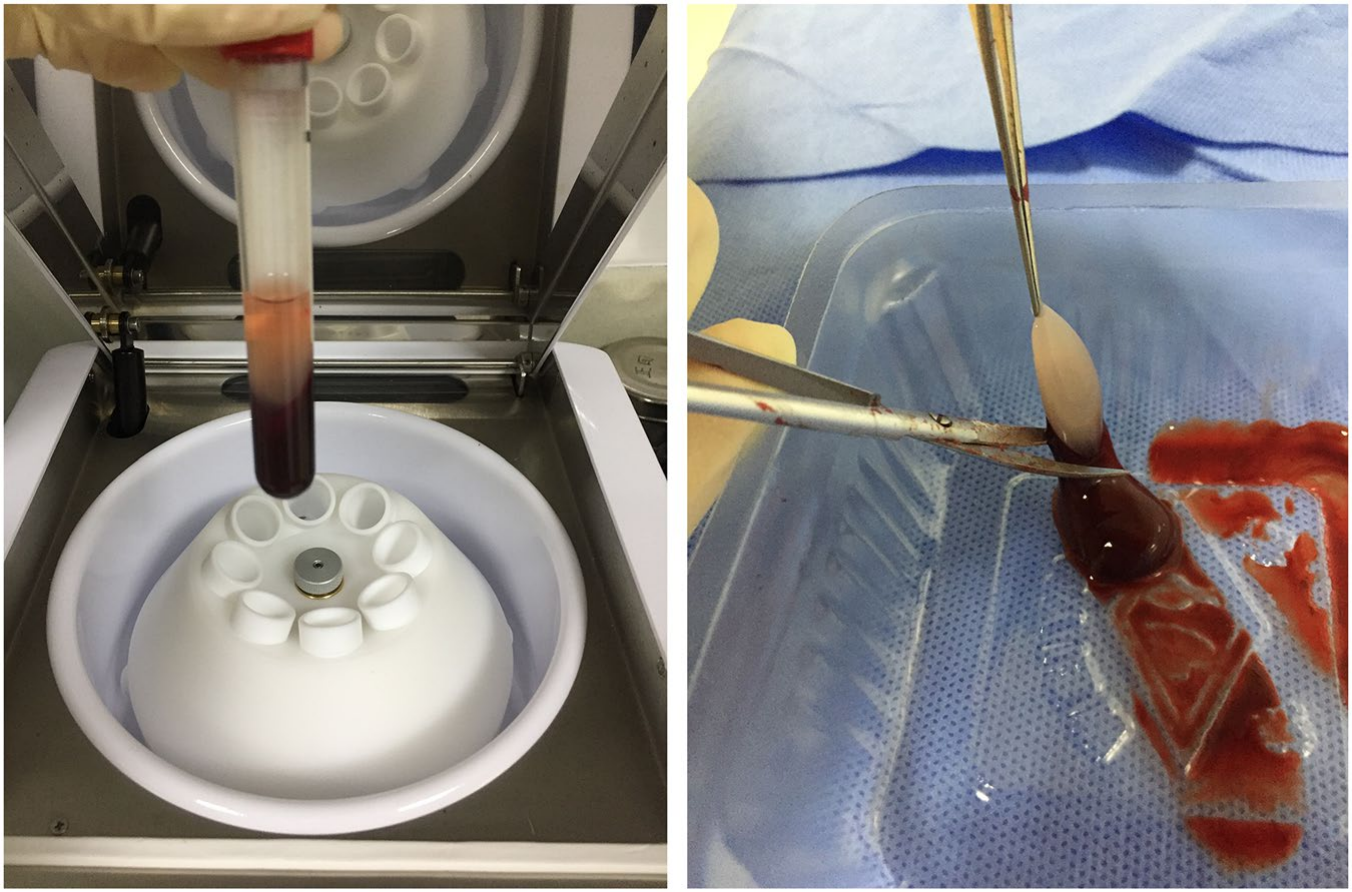
CGF was prepared from rat blood without anticoagulants by centrifuging in a special centrifuge device, then used for the *in vivo* experiments.

For the *in vivo* experiments, CGF was obtained from the heart blood of healthy male Wistar rats (age 8 weeks). Donor rats were anesthetized by intraperitoneal injection of 10% chloral hydrate (0.4 ml/100 g); blood was taken by cardiac puncture directly into a sterile tube without anticoagulants and was immediately centrifuged in a special centrifuge device (Medifuge^TM^; Silfradent srl, Sofia, Italy) for approximately 13 minutes. CGF, the fibrin buffy coat used in this *in vivo* study, was separated using scissors (Fig. 1). The three-dimensional (3D) structures of CGF and BMSCs cultured with CGF for 7 days were observed by scanning electron microscopy (SEM, JEOL, Japan).

### *In vivo* reconstruction of calvarial defects in rats

**Figure 2:**
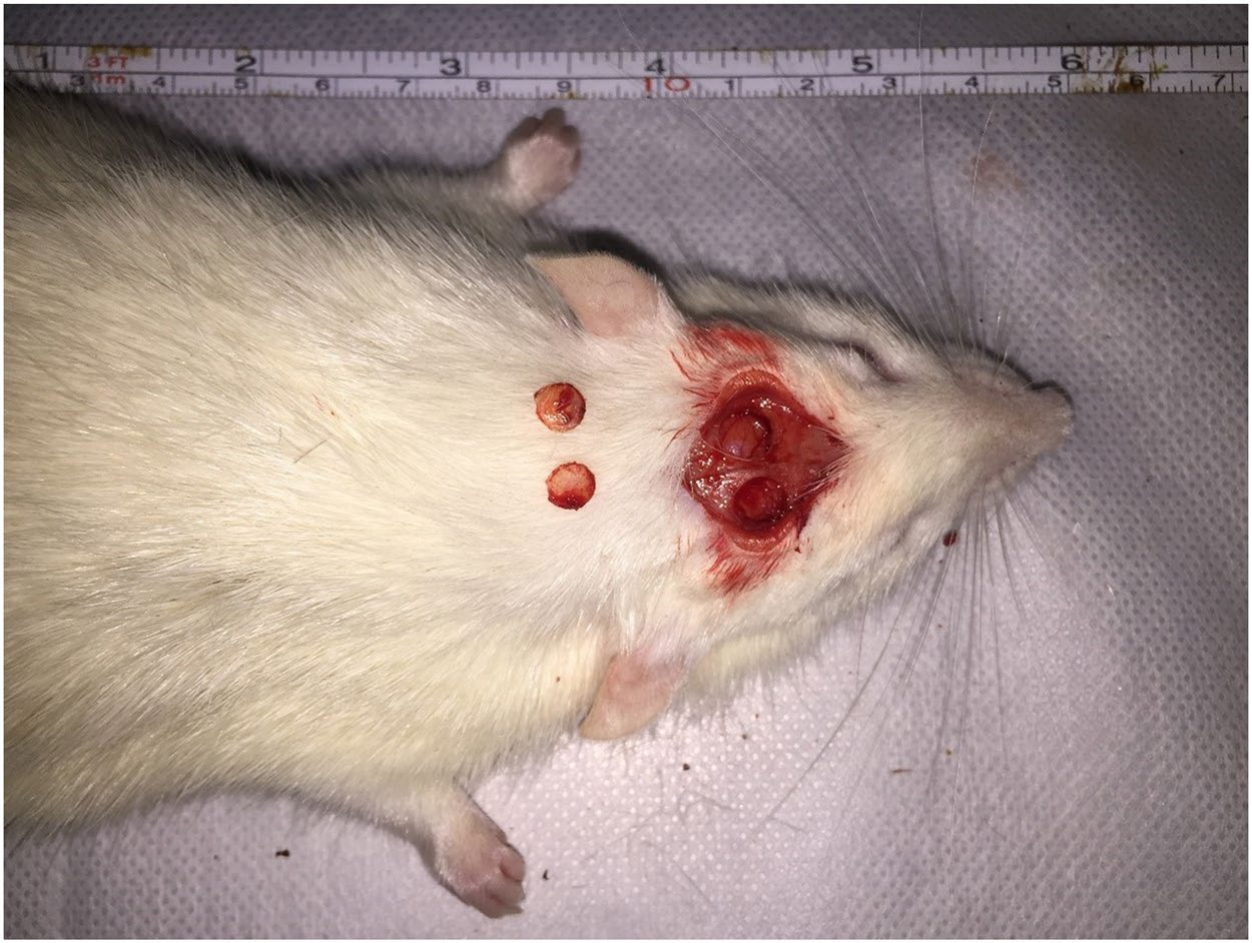
Two bilateral critical-sized calvarial defect models without perichondrium were created.

Thirty male Wistar rats were divided randomly into five calvarial defect groups. The rats were anaesthetized by intraperitoneal injection of 10% chloral hydrate (0.4 ml/100 g), a 1.5 cm sagittal incision was made in the scalp, and then the calvarium was exposed by blunt dissection. Two bilateral critical-sized defects without perichondrium were created (Fig. 2) using a 5.5-mm diameter trephine burr (Medesy, Italy). In the experimental group, we used BMSCs that had been initially cultured with CGF for 7 days *in vitro* and then transferred *in vivo*. The collagen used as control scaffolds in the study was type I rat tail tendon collagen (Shengyou, China). Non-filled calvarial defects served as blank controls. Finally, sixty critical-sized calvarial defects in thirty Wistar rats were randomly filled as follows: (A) CGF + BMSCs; (B) CGF; (C) collagen + BMSCs; (D) collagen; (E) blank.

### Microcomputed tomography (Micro-CT) measurement

The samples were scanned with a micro-CT system (uCT-80, Scanco, Switzerland) using 10 μm spot size and 55 KVp maximum voltage parameters to determine the amount of newly formed bone. 3D images were generated.

### Histological and immunohistological observation

The specimens were decalcified in 10% EDTA for two weeks after being examined by micro-CT, dehydrated in 75% to 100% ascending alcohol concentrations, and then embedded in paraffin. Histological sections were prepared for Hematoxylin and Eosin (H&E), Masson staining and immunohistological analysis. The primary antibodies CD34 (1:1000 dilution; Servicebio) and the secondary antibody (1:1; DAKO) were successively subjected to immunohistological assay. The samples were then observed and photographed under a high quality microscope.

### Statistical analysis

Group statistical analysis was performed with ANOVA using SPSS v.10.1 software (SPSS Inc, USA). Data results were expressed as means ± SD. Values were considered statistically significant at p < 0.05.

## Results

### Flow cytometric identification of BMSC surface markers

**Figure 3:**
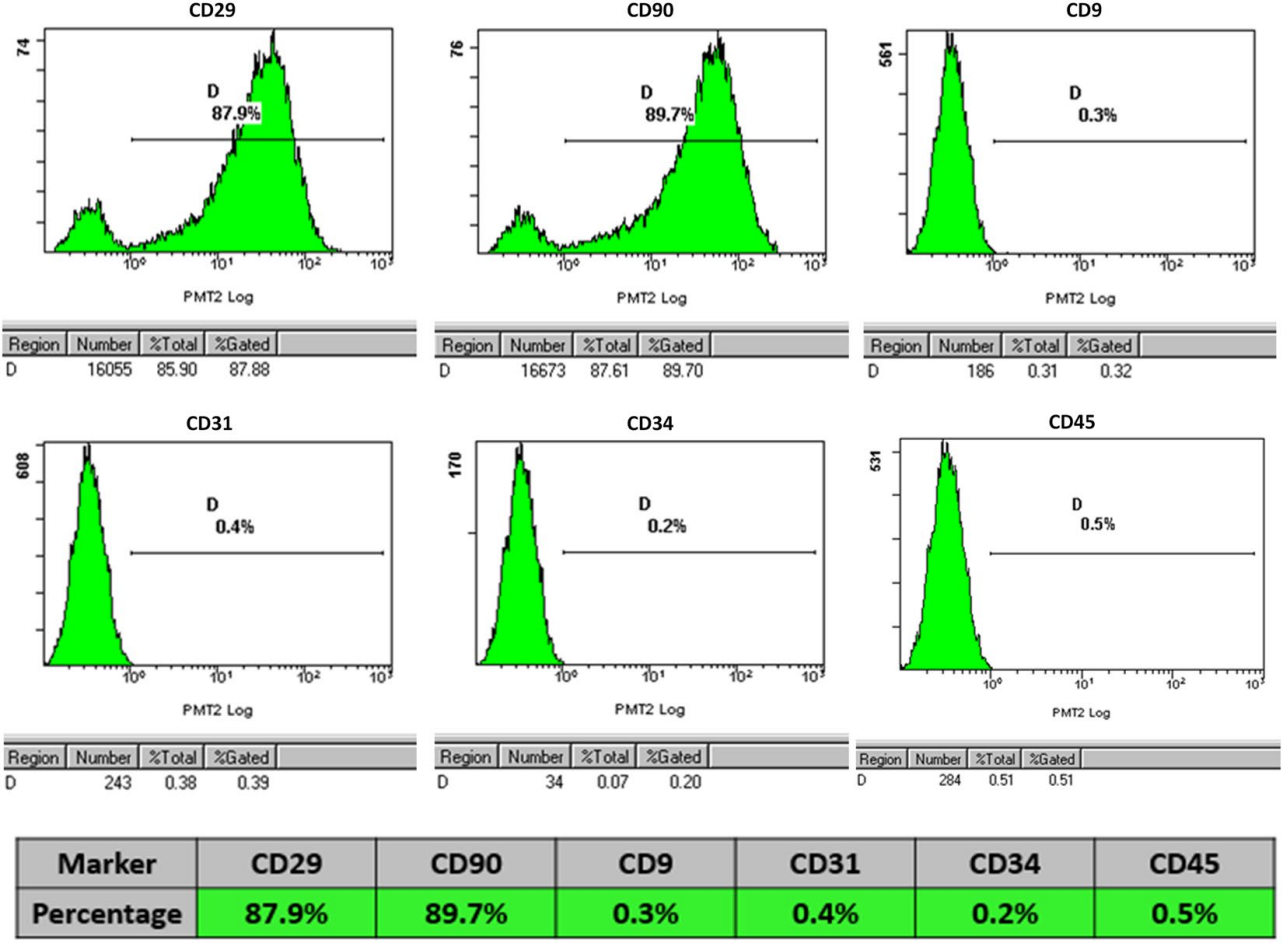
Cytometric flow analysis indicated that the cells in the research were negative for CD9, CD31, CD34 and CD45 but expressed the positive mesenchymal-associated markers CD29 and CD90.

The primary and passaged BMSCs had normal morphologic characteristics and typical fibroblast-like colony forming distribution. Flow cytometric analysis revealed the isolated and cultured cells were positive for the MSC markers CD29 (87.9%) and CD90 (89.7%) but negative for CD9 (0.3%), CD31 (0.4%), CD34 (0.2%) and CD45 (0.5%), respectively (Fig. 3).

### Material characterization

**Figure 4:**
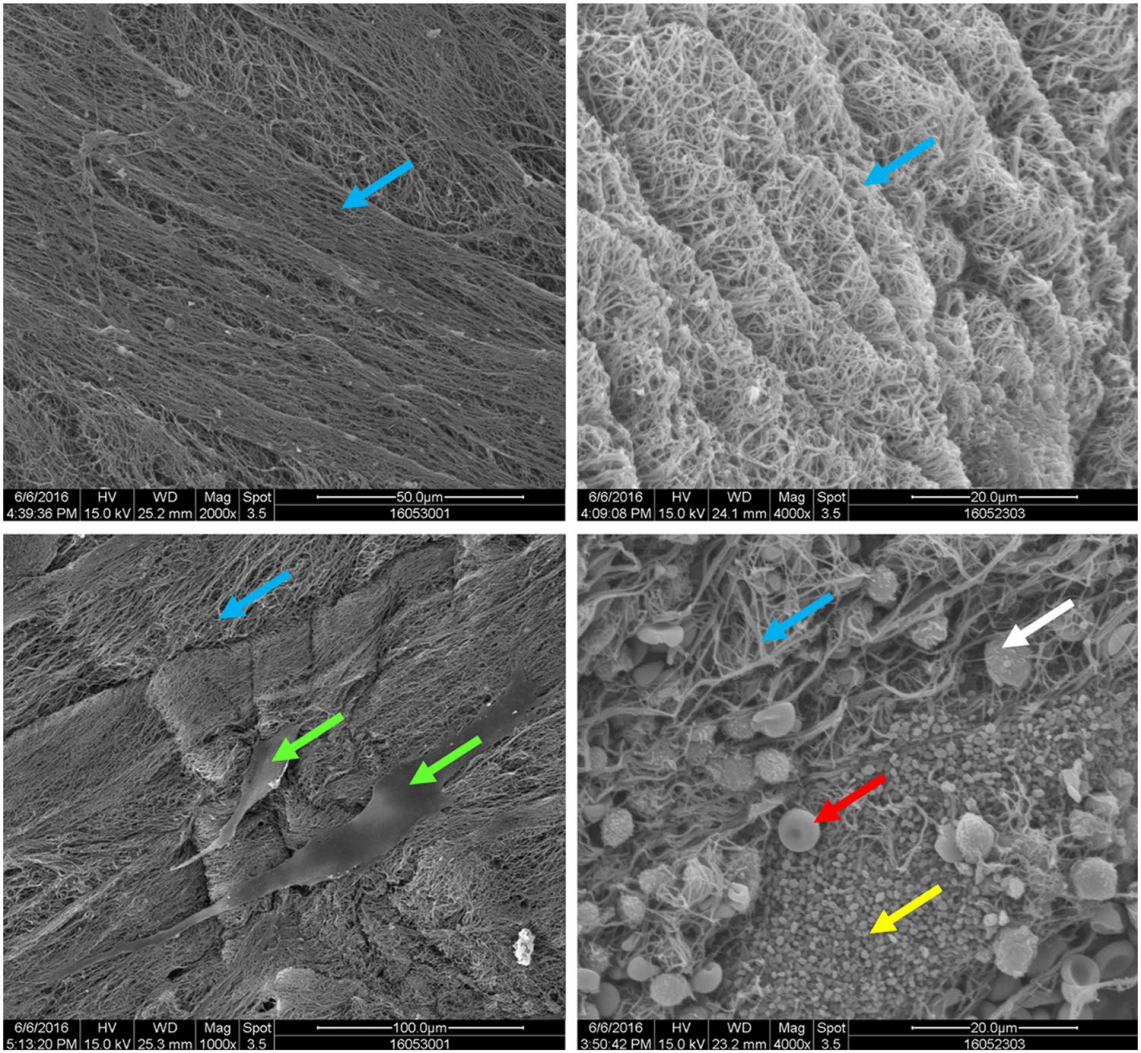
The ultrastructure of the CGF (scanning electron microscopy observation). The 3-dimensional network consisted predominantly of fibrin fibers (blue arrow). BMSCs can be observed growing well on the biomaterial surface (green arrow). Numerous platelets (yellow arrow), leukocytes (white arrow) and red blood cells (red arrow) are embedded in the network.

The SEM images of CGF scaffolds cultured with BMSCs for 7 days are presented in Fig. 4. The images show that the samples have regular fibrinogen structures and contain white blood cells, red blood cells and blood platelets. BMSCs can be clearly seen growing well on the surface of the CGF.

### *In vivo* regeneration of calvarial defects of rats

**Figure 5:**
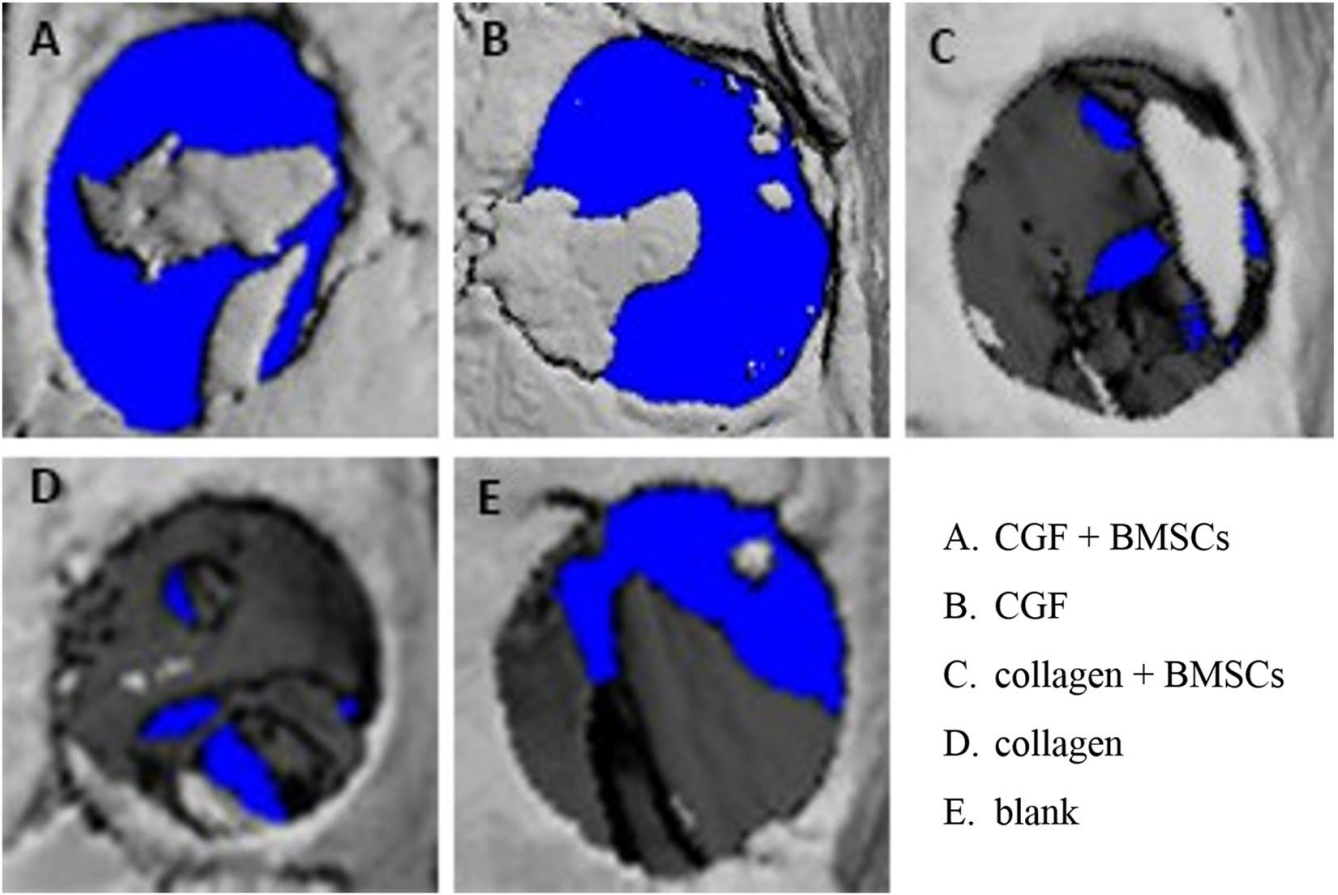
3D images of the newly formed bone 6 weeks after operation were reconstructed by micro-CT in five different experimental groups: (**A**) CGF + BMSCs; (**B**) CGF; (**C**) collagen + BMSCs; (**D**) collagen; (**E**) blank.

**Figure 6:**
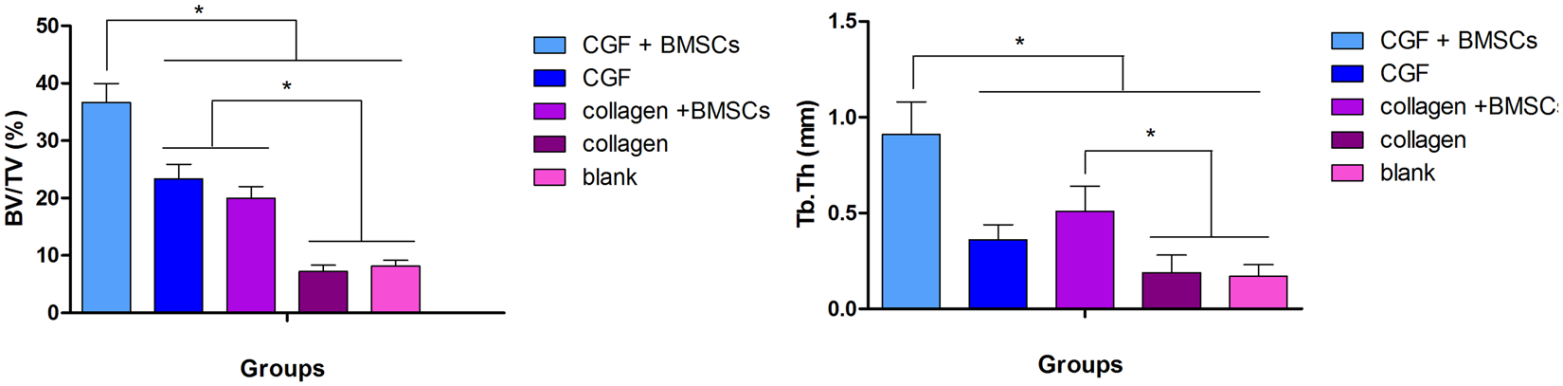
New bone formation analysis of BV/TV and Tb.Th among five groups at 6 weeks postoperation. *Indicates significant differences between each groups, p < 0.05.

Figure 5 shows newly formed bone 6 weeks after operation reconstructed by micro-CT. The (A) CGF + BMSC group showed the highest level of bone regeneration, while the (B) CGF and (C) collagen + BMSC groups outperformed the (D) collagen and (E) blank groups. Morphometrical analysis showed significantly greater BV/TV and trabecular thickness (Tb.Th) in the (A) CGF + BMSC group (36.70 ± 3.27% and 0.91 ± 0.17 mm, respectively) compared with the (B) CGF group (23.34 ± 2.57% and 0.36 ± 0.08 mm, respectively), the (C) collagen + BMSCs group (19.98 ± 2.03% and 0.51 ± 0.13 mm, respectively), the (D) collagen group (7.23 ± 1.08% and 0.19 ± 0.09 mm) and the (E) blank group (8.18 ± 0.97% and 0.17 ± 0.06 mm, respectively) (p < 0.05) (Fig. 6).

### Histological and immunohistological analysis of bone regeneration

An osteogenic histological assessment of the five groups was carried out 6 and 12 weeks after the rat calvarial defect operation. Bone sections that had undergone H&E and Masson staining were examined under a microscope. Histological results revealed different bone regeneration results in the different groups. In the (D) collagen and (E) blank control groups, almost no new bone was found in the defect site 6 weeks postoperatively and the fibrous connective tissue over the empty defects was sparse. This pattern of bone formation was also observed after 12 weeks; a large bone defect still remained in these two groups.

In the (B) CGF and (C) collagen + BMSCs groups, at 6 weeks postoperatively, fibrous connective tissue was observed over the defects and some new bone had formed along the boundary of the bone defect. In contrast with the (D) collagen and (E) blank groups, Masson staining showed matrix collagen fibrils were widely distributed in the (B) CGF and (C) collagen + BMSC groups. The new bone grew gradually from the bone defect boundary to the central area. Twelve weeks after operation, newly formed bone covered almost half the bone defect area. The difference between the (B) CGF and (C) collagen + BMSC groups was that neovascularization was observed in histological sections of the (B) CGF group.

**Figure 7:**
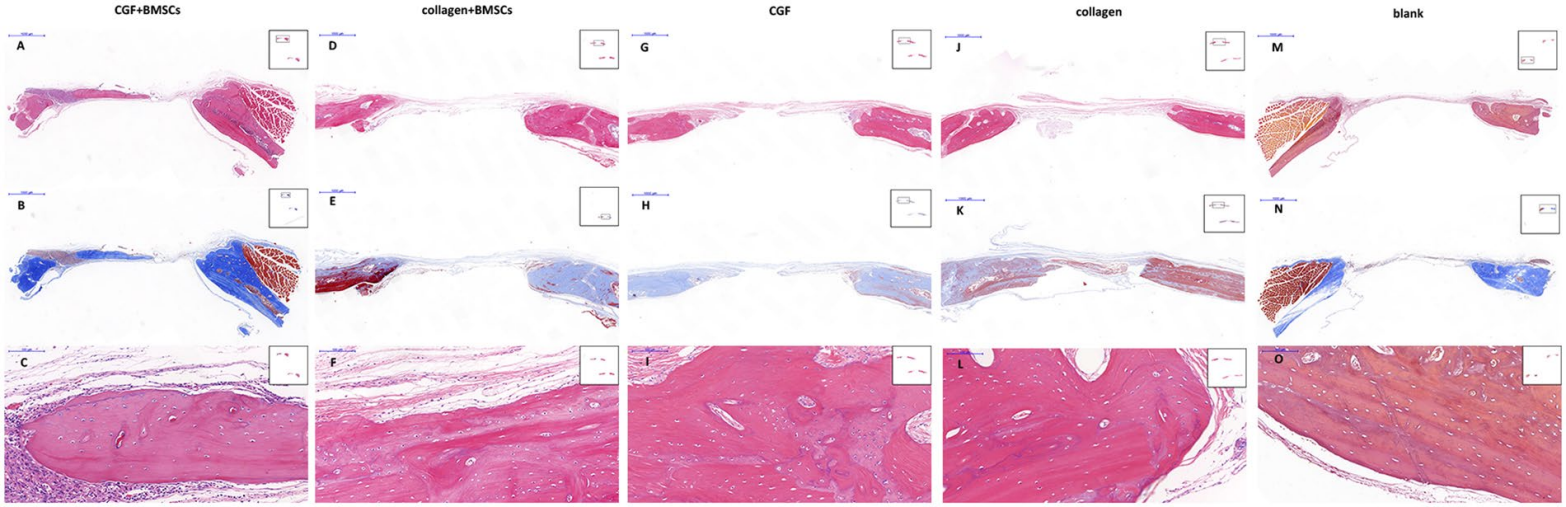
The microscopic histological images of cranial tissue sections of different groups, stained with H&E (**A**,**C,D,F,G,I,J,L**,**M**,**O**) and Masson (**B**,**F**,**H**,**K**,**N**) 6 weeks postoperatively. Newly formed bone was found both in the center and periphery of the defect site in the CGF + BMSCs group (**A**,**B**), this phenomenon is not observed in other groups (magnification × 20; bar 1,000 um). Neovascularization was observed around the new bone in the CGF + BMSCs group (**C**), and the structure of newly formed bone is the same as normal bone structure (**C**,**F**,**I**,**L**,**O**) (magnification × 200; bar 100 um).

**Figure 8:**
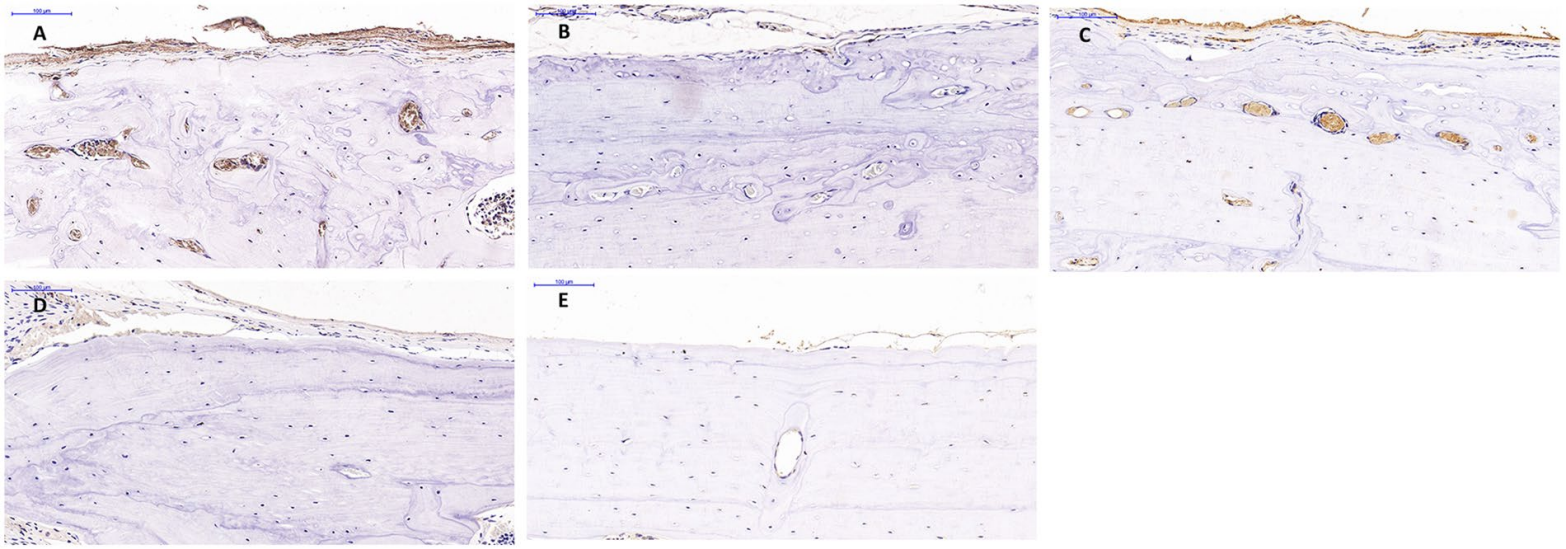
The images (magnification × 200; bar 100 um) show the CD34 immunohistochemistry (IHC) results. Contrary to the (**B**) collagen + BMSCs, (**D**) collagen and (**E**) blank groups, neovascularization was observed in the (**A**) CGF + BMSCs group and also in the (**C**) CGF group. However, neovascularization in the (**C**) CGF group was less than in the (**A**) CGF + BMSCs group.

In the (A) CGF + BMSC group 6 weeks postoperatively, newly formed bone was found both in the center and the boundary of the defect site, with wide distribution of the collagen fibrils of the matrix (Fig. 7). Micro-CT images confirmed the islands of newly-formed bone in contrast with the other groups. Distinct neovascularization around the new bone was observed in histological analysis and confirmed by CD34 immunohistology (Fig. 8). The regenerated bone gradually matured. 12 weeks postoperatively, the defect was almost filled by new bone. The newly formed bone had the same histological structure as normal bone.

## Discussion

Autogenous bone grafting or artificial materials can be used to reconstruct bone defects, but reducing bone absorption and enhancing new bone formation has remained problematic; the risk of bioincompatibility, infection and immunological rejection are high both for allogenic bone grafting and artificial materials. Tissue engineering technology may make bone defect reconstruction feasible. In some clinical studies, autologous materials acting as a biological scaffold were used to ensure graft stabilization; satisfactory long-term results were obtained^16^. For example, Hibi *et al*. applied PRP and autologous mesenchymal stem cells to repair the alveolar cleft of a 9-year-old female patient and regenerated bone was observed by CT after three months with 79.1% remaining after nine months^17^.

In our experiment, the properties of bone marrow-derived mesenchymal stem cells were characterized using cell surface markers. The detection of surface molecule expression revealed that BMSCs were negative for CD45, CD34, CD31 and CD9 but positive for mesenchymal associated markers such as CD90 and CD29. The BMSCs played a key role in the bone regeneration^6^.

In the present study, we compared the effects of different groups – CGF or collagen with or without BMSCs – which have not been discussed previously in the literature. CGF, the third-generation platelet concentrate, has a higher GF concentration and better regenerative capacity than PRF (the second-generation platelet concentrate) and PRP (the first-generation platelet concentrate)^18,19^. Our study confirms the positive effects of a combination of bone marrow stromal cells and CGF in an experimental bone defect model. In our study, micro-CT and histological examination found new bone formation not only at the periphery of the bone defect, but also in the central area of the bone hole in the (A) CGF + BMSC group, starting 6 weeks after the operation. Newly formed bone appeared not only at the periphery, but also in the centre of the bone defect. Other groups had a gradual progression of new bone from the bone defect periphery to the center. New bone grew in this direction because the cells that induce bone formation were mainly supplied from the cut edge of the bone defect. The different phenomenon in (A) CGF + BMSC group showed that CGF provides a three-dimensional scaffold for BMSCs to survive and grow continuously and steadily. After twelve weeks, critical-size bone defects were almost closed over in the (A) CGF + BMSC group, whereas large bone defects remained in the (D) collagen and (E) blank control groups. In the (B) CGF and (C) collagen + BMSC groups, new bone grew slowly. These results demonstrate that the CGF fibrin buffy coat is a biomaterial that can both bear BMSCs and secrete growth factors and thereby promote new bone formation, as reported by Honda *et al*^20^. By making comparisons between five different groups – CGF + BMSCs, collagen + BMSCs, CGF, collagen and blank group – we were able to identify the most powerful osteogenesis effect while excluding BMSC interference. In addition, compared to the other groups, starting six weeks postoperatively, new bone mass significantly increased in the (A) CGF + BMSC and (C) collagen + BMSCs groups, and this tendency continued for 12 weeks, confirming the importance of bone marrow-derived stem cells.

Not only does CGF provide a three-dimensional fibrin scaffold structure for BMSCs, it releases multiple growth factors which regulate cell proliferation and differentiation via specific receptors^21^. Vascular endothelial growth factor (VEGF) stimulates angiogenesis, an important step in the bone regeneration process because the supply of blood favors osteogenesis^22^. It has been shown that angiogenesis occurs before osteogenesis in the healing of bone defects, and that angiogenesis and osteogenesis have a mutually reinforcing effect on bone regeneration^23^. Given that angiogenic factors play an important role both in healing and in regeneration^24^, VEGF may induce mobilization, recruitment, proliferation and differentiation of endothelial progenitor cells (EPC), as well as the recruitment and survival of osteoblasts^25,26^. Our histological and immunohistochemical analysis showed that CGF promotes neovascularization better than groups without CGF. Neovascularization may enhance impaired angiogenic capacity and facilitate bone healing. Based on these results, we believe that CGF may induce osteogenic differentiation of BMSCs as well as angiogenesis.

Combination CGF/BMSC therapy has the full panoply of osteogenesis tissue engineering advantages – osteogenic seed cells: BMSCs, cell-bearing scaffold: CGF fibrin network, osteoinductive and osteoconductive cytokines: concentrated growth factors – that favour effective bone growth and regeneration. Preparing CGF and cells cultivated with CGF is quick and easy. Most importantly, CGF is autologous, spontaneously degraded, and absolutely safe. These *in vivo* results indicate that BMSC-stimulating CGF is an ideal biological material for bone regeneration.

## Conclusions

This study demonstrates that CGF not only promotes the superior osteoinductive activity of BMSCs to enhance bone formation, but also outperforms collagen in stimulating angiogenesis. We conclude that CGF is an excellent cell growth factor biomaterial. Combined with BMSCs, it has a strongly positive effect on both osteogenesis and angiogenesis, making it a very promising material for bone regeneration.
